# Characterization of polymorphic microsatellite loci for North American common greenbrier, *Smilax rotundifolia* (Smilacaceae)

**DOI:** 10.1002/aps3.1163

**Published:** 2018-06-26

**Authors:** Ruihong Wang, Mengdi Li, Xue Wu, Chao Shen, Wendi Yu, Jinliang Liu, Zhechen Qi, Pan Li

**Affiliations:** ^1^ College of Life Sciences Zhejiang Sci‐Tech University Hangzhou 310058 People's Republic of China; ^2^ Zhejiang Province Key Laboratory of Plant Secondary Metabolism and Regulation Hangzhou 310018 People's Republic of China; ^3^ The Key Laboratory of Conservation Biology for Endangered Wildlife of the Ministry of Education, and Laboratory of Systematic and Evolutionary Botany and Biodiversity College of Life Sciences Zhejiang University Hangzhou 310058 People's Republic of China

**Keywords:** microsatellites, North America, phylogeography, Smilacaceae, *Smilax rotundifolia*, transcriptome

## Abstract

**Premise of the Study:**

Microsatellite markers were developed for *Smilax rotundifolia* (Smilacaceae), an understory vine widely distributed in eastern North America, to investigate genetic diversity and structure. Cross‐amplification was tested in three congeneric species: *S. china*,* S. riparia*, and *S. walteri*.

**Methods and Results:**

A total of 6153 simple sequence repeat primer pairs were detected from the de novo–assembled transcriptome data (88,3962 contigs) of *S. rotundifolia*. Thirty‐three polymorphic microsatellite loci were selected for further analysis among 96 individuals representing four natural populations of the species. The number of alleles ranged from two to 15, and 87.9% of the developed primer pairs could be cross‐amplified in at least one of three congeneric *Smilax* species.

**Conclusions:**

The simple sequence repeat markers developed in this study will facilitate further studies on genetic diversity and phylogeographic patterns of *S. rotundifolia* and provide additional potential microsatellite resources for other *Smilax* species.

*Smilax rotundifolia* L. (Smilacaceae), the common greenbrier, is a woody, dioecious understory vine characterized by shiny, roundish, heart‐shaped leaves, frequently four‐sided stems, broad prickles, and black glaucous berries. It is a typical element of temperate and subtropical forests in eastern North America, where it is distributed extensively (Holmes, 2002). Molecular markers such as microsatellites could examine its range‐wide population genetic structure and possibly predict how historical processes (e.g., climate oscillations, habitat change, post‐glacial migration) have shaped its current wide distribution in North America. Although microsatellite markers have been previously developed for *S. aspera* L. and *S. sieboldii* Miq. (Qi et al., 2017; Ru et al., 2017), these showed low transferability (<30%) to *S. rotundifolia*. This may be because these species belong to three deeply divergent (ca. 40–25 mya) major clades of Smilacaceae: *S. aspera* in the Old World basal clade, *S. sieboldii* in the New World B clade, and *S. rotundifolia* in the New World A clade (Qi et al., 2013; Chen et al., 2014). To further study the phylogeography of *S. rotundifolia* in North America, here we developed 33 new variable microsatellite markers based on transcriptome data. Moreover, to broadly test their transferability within the family, three congeneric species (*S. china* L. in the Old World D clade, *S. riparia* A. DC. in the Old World C clade, and *S. walteri* Pursh in the New World B clade) from three major clades of Smilacaceae were selected according to the phylogeny of Qi et al. (2013).

## METHODS AND RESULTS

### Plant sample collection, DNA extraction, and transcriptome sequencing

A total of 96 samples of *S. rotundifolia* from four populations (24 individuals per population) were collected to develop and validate microsatellite primers for this species. Three congeneric species (*S. china*,* S. riparia*, and *S. walteri*) were selected for tests of cross‐amplification of the markers. A total of 15 individuals, including five individuals from each of the three species, were sampled. Detailed information on all the samples is provided in Appendix 1. Total genomic DNA was extracted from silica gel–dried leaf materials using the Plant DNAzol Kit (Thermo Fisher Scientific, Waltham, Massachusetts, USA). Total RNA was extracted from young leaves of *S. rotundifolia* using a cetyltrimethylammonium bromide (CTAB) procedure (Chang et al., 1993). The poly(A)+ RNA (mRNA) was purified and fragmented into small pieces (200 bp) by NEBNext First Strand Synthesis Reaction Buffer (5×) (New England Biolabs, Boston, Massachusetts, USA). First‐strand cDNA was synthesized using a random hexamer primer and M‐MuLV Reverse Trancriptase (RNase H) (New England Biolabs). Second‐strand cDNA synthesis was subsequently performed using DNA polymerase I and RNase H. Illumina TruSeq Stranded mRNA paired‐end sequencing adapters (Illumina, San Diego, California, USA) were then ligated to the ends of the 3′‐adenylated cDNA fragments. The index‐coded cDNA library was sequenced by Novogene Co. Ltd. (Beijing, China) on the Illumina HiSeq 4000 platform, yielding 150‐bp paired‐end reads. Before assembly, raw reads were filtered using Trimmomatic (Bolger et al., 2014) to remove adapter sequences, low‐quality reads (>20% of nucleotides with *Q* value ≤ 10), and reads containing >10% ambiguous base calls. The raw sequencing data were submitted to the National Center for Biotechnology Information (NCBI; BioSample accession SAMN08329984). Transcriptome assembly was performed using Trinity version 2.5 with the default parameters (Grabherr et al., 2011).

### Development of SSR markers

A total of 88,962 contigs were prepared for simple sequence repeat (SSR) targeting and primer design using MIcroSAtellite identification tool (MISA; Thiel et al., 2003). SSR searches were performed with motifs ranging from mono‐ to hexanucleotides, and 6153 primer pairs were designed using Primer3 software (Rozen and Skaletsky, 1999) with default settings. One hundred and thirty primer pairs were randomly chosen for preliminary testing using four samples (one individual per population) of *S. rotundifolia* to ensure the availability and optimal annealing temperature of each pair. A gradient PCR amplification was performed, with the reaction mixture containing 30 ng of genomic DNA, 5 μL of 2× Master Mix (Tsingke Biotech Co., Hangzhou, Zhejiang, China), 0.2 μM of forward and reverse primers, and ddH_2_O to reach a volume of 10 μL. The PCR procedure was run as follows: initial denaturation at 94°C for 5 min; followed by 30 cycles at 94°C for 45 s, a temperature gradient for annealing from 50°C to 65°C for 45 s, and 72°C for 1 min; and a final extension at 72°C for 5 min. Forty primer pairs with products showing a clear single band on agarose gel were selected for further tests of variation and transferability.

### Polymorphism and transferability assessment

To screen polymorphisms for these 40 loci, we genotyped 96 individuals from four populations of *S. rotundifolia* using a two‐step PCR amplification protocol (Schuelke, 2000; Sakaguchi and Ito, 2014). In the first step, the PCR reaction was performed following the procedures described above, with the following exceptions: we used 0.1 μM of sequence‐specific forward primer with an M13 tail at its 5′ end, 0.4 μM of sequence‐specific reverse primer, and a 45 s annealing time, with the annealing temperature set to the optimal temperature of the specific primers used (see Table 1). In the second step, the products of the first step were used, with 5 μL of 2× Master Mix, another 0.8 μL (5 μM) of fluorophore‐labeled universal M13 primer (FAM, ROX, HEX, or TAMRA), and additional ddH_2_O to reach a final volume of 20 μL. The PCR procedure was as follows: initial denaturation at 94°C for 3 min; 15 cycles at 94°C for 30 s, annealing at 53°C for 30 s, and 72°C for 45 s; and a final extension at 72°C for 10 min. PCR products were separated on an ABI PRISM 3730xl Genetic Analyzer with GeneScan 500 LIZ internal size standard (Applied Biosystems, Waltham, Massachusetts, USA). Geneious version 9.0.2 (Kearse et al., 2012) was applied to score genotypes. Finally, 33 polymorphic primer pairs were selected for transferability and further population genetic study (Table 1).

**Table 1:** Characteristics of 33 microsatellite loci developed for *Smilax rotundifolia*

Locus	Primer sequences (5′–3′)	*A*	Repeat motif	Allele size range (bp)	*T*_a_ (°C)	GenBank accession no.
D004	F: GATTCTTCAAGACCGACCCA	6	(GA)_8_	110–158	50	MG645948
	R: GAGCGGGAATCCATACAAGA					
D005	F: TGTACAAGAGGAAAAGGAAACCTC	5	(GA)_6_	180–192	51	MG645949
	R: CCTTTTGGCACCTATGGAGA					
D007	F: GCACGAACAGTATGAAGCGA	15	(TC)_6_	121–149	50	MG645950
	R: AGGAGAGAGAGGAGAACGGC					
D009	F: AACGCATGCATTCAAACAAG	10	(TG)_10_	168–186	50	MG645951
	R: AGCCTCATGCACTGGACTGT					
D010	F: ATAGGCAGGGAAAGGAGGAA	6	(TC)_6_	178–186	50	MG645952
	R: GAGGGAGAGCTAAAGCCCAT					
D012	F: AATCTTTTGGGGTAGGTGGG	6	(CGT)_6_	173–191	50	MG645953
	R: TGAAATAGAGAGTGCGGCCT					
D014	F: GAGCGACATCTTTCCCTCAG	4	(CTC)_6_	177–186	50	MG645954
	R: GCCCTCTTCGAATCCCTAAC					
D016	F: GCTATTGGTGTGGTTCAGGG	3	(GGT)_6_	189–195	59	MG645955
	R: GCCTCGTCTTCTTCCTCCTC					
D017	F: AAGAGGAGCTGGAGGACGAC	2	(AGA)_5_	180	59	MG645956
	R: CAGCATTCTCCTTTTCCTGC					
D021	F: TATCCTTTGCCGAACGAAAG	7	(TGC)_6_	204–225	51	MG645957
	R: GGGGCAGGGGTTTGATAATA					
D023	F: CCAAAATGGTGTTGCTGTTG	2	(GAA)_5_	129–132	50	MG645958
	R: AGCAAAGACAGGTGCTCCAT					
D024	F: GGTATAGGGATGCGAGTCCA	4	(AGAT)_5_	158–170	51	MG645959
	R: TTTCTGACGAACATTGAGCG					
D025	F: TATGTATGCCCATCCATCCA	6	(GGAA)_5_	126–150	50	MG645960
	R: CAGGTCTGGAAAACGAGGAG					
D027	F: CGTCCTTTGTTTCACGCTTT	3	(TCGA)_5_	130–138	51	MG645961
	R: CAAACAGGTTGAGGGCAACT					
D029	F: GGAGTGTGGATTTGTGGCTT	6	(CCTT)_6_	178–198	50	MG645962
	R: CATCCCCCAACAAGAGCTAA					
D032	F: GACGACCTTCTGCTTCTTGG	5	(CGAT)_5_	214–254	54	MG645963
	R: AAAACCCCAAACTCCAAACC					
D033	F: TGTTTGATTGAGGAGAGGGG	7	(ATCC)_5_	188–212	50	MG645964
	R: TTTGGCCCTGAGCAATTTAT					
D036	F: TAAATTCCAAAGGAGCACGC	6	(ATTT)_5_	158–178	51	MG645965
	R: TTCTGACCCTCCACCCATAG					
D038	F: TTGCTGACTTCACCAGCATC	5	(CTTCC)_7_	159–179	50	MG645966
	R: TCTCTCCCGAGTTCCTCTGA					
D039	F: CTTGGAGAGGGATGGGTACA	5	(ATGTT)_5_	221–236	50	MG645967
	R: CCCTTGTACAAAACAAAGGCA					
R007	F: TCCGTGCTCTCACTTCCTCT	7	(TC)_6_…(CT)_6_	200–236	51	MG645968
	R: GATTGGTTTTGGGAGTTGGA					
R022	F: ACAACGGCCAGATTTGTTTC	8	(CT)_9_	182–198	50	MG645969
	R: ACACAAAGGGAGTGGTTTGG					
R026	F: CATCCCTGTCCGCTTAACAT	5	(AG)_8_	323–331	50	MG645970
	R: CGCTCTTGAGGGTGTAGGAG					
R031	F: GAGCTTGGCCTTTCAAAGAA	6	(CA)_7_	257–269	56	MG645971
	R: GGAAGTGGCACGAGGTATGT					
R037	F: TACTCCTACACGCCTCCCAC	10	(TC)_7_	124–160	50	MG645972
	R: CTGGGATTGGGATTGAGAGA					
R043	F: GCCGTGTTTCTATTTCGAGG	13	(CCT)_5_	141–180	50	MG645973
	R: TCGTGGGTGAGGAGAGAGAG					
R045	F: GTAAACCTGGCACCGAAGAA	8	(CTG)_5_	305–344	60	MG645974
	R: CACTTCCCAGATCCCTACCA					
R049	F: TCAAATGCAGCTCAATCAGG	8	(AAG)_6_	232–256	50	MG645975
	R: CCCACTGATAACTGCCCTCT					
R054	F: TGCTGGAATCACTGTTTTCG	3	(ATC)_5_	345–351	56	MG645976
	R: AGCCTCAACTTCCATCCCTT					
R056	F: TGTAGGTCTTGGAGAACGGG	4	(TCG)_5_	116–125	50	MG645977
	R: TTTGTGGATGGACTTGGACA					
R058	F: TTCTCTCCATCATCGCCTCT	10	(CAT)_6_	109–181	50	MG645978
	R: GCTCCACCTCCTTCCCTATC					
R087	F: ATGGTGTGGATGGAGGTGTT	8	(TCGTGC)_6_	124–178	50	MG645979
	R: AGCGGTGAAGTGGATGCTAT					
R090	F: AGCCGAACTTCTTGGACTCA	4	(CCGCGT)_5_	286–304	50	MG645980
	R: GTACAAGCGAGTAAAGGCGG					

Note

*A* = number of alleles per locus; *T*_a_ = optimized annealing temperature.

Number of alleles, observed and expected heterozygosity, polymorphism information content, and deviations from Hardy–Weinberg equilibrium were calculated and tested using CERVUS 3.0 (Kalinowski et al., 2007). For each population of *S. rotundifolia*, the number of alleles ranged from two to 15, polymorphism information content ranged from 0.011 to 0.813, and the levels of observed and expected heterozygosity varied from 0.011 to 0.958 and 0.011 to 0.836, respectively. The Hardy–Weinberg equilibrium test indicated that three primer pairs deviated significantly from expected values (Table 2). In cross‐amplification tests, a 1.5% agarose gel was used to detect the transferability of each primer, and the observation of one clear distinct band in the expected size range was considered as successful amplification. The transferability rate of each species was 57.6% in *S. riparia* and *S. china*, and 69.7% in *S. walteri*. In total, 87.9% SSR primers were successfully cross‐amplified in at least one species (Table 3).

**Table 2:** Genetic parameters of the 33 microsatellite loci within four populations of *Smilax rotundifolia*.^a^

Locus	CE (*N* = 24)				CS (*N* = 24)				NE (*N* = 24)				SE (*N* = 24)			
	*A*	*H*_o_	*H*_e_	PIC^b^	*A*	*H*_o_	*H*_e_	PIC^b^	*A*	*H*_o_	*H*_e_	PIC^b^	*A*	*H*_o_	*H*_e_	PIC^b^
D004	4	0.542	0.680	0.600	3	0.348	0.537	0.426	5	0.500	0.577	0.473	4	0.917	0.629	0.552*
D005	3	0.292	0.370	0.310	3	0.333	0.677	0.589	3	0.217	0.563	0.477	4	0.174	0.532	0.440
D007	7	0.696	0.803	0.755	7	0.913	0.804	0.754	8	0.364	0.796	0.752	11	0.739	0.798	0.750
D009	8	0.636	0.829	0.783	7	0.304	0.777	0.724	7	0.583	0.812	0.765	8	0.706	0.870	0.825
D010	3	0.095	0.463	0.385	2	0.286	0.254	0.215	6	0.130	0.550	0.506	3	0.059	0.508	0.397
D012	4	0.667	0.686	0.607	5	0.238	0.659	0.576	5	0.444	0.751	0.683	5	0.400	0.727	0.653
D014	3	0.417	0.630	0.544	3	0.250	0.551	0.432	2	0.333	0.383	0.305	4	0.750	0.618	0.523
D016	1	0.000	0.000	0.000	2	0.048	0.048	0.045	2	0.000	0.082	0.077	1	0.000	0.000	0.000
D017	1	0.000	0.000	0.000	1	0.000	0.000	0.000	2	0.042	0.042	0.040	1	0.000	0.000	0.000
D021	4	0.391	0.403	0.363	5	0.833	0.781	0.729	6	0.500	0.582	0.522	5	0.522	0.525	0.483
D023	2	1.000	0.511	0.375***	2	0.958	0.510	0.375**	2	1.000	0.511	0.375***	2	0.875	0.503	0.371
D024	2	0.214	0.198	0.173	3	0.500	0.558	0.468	3	0.278	0.446	0.386	4	0.118	0.586	0.484
D025	3	0.435	0.498	0.432	6	0.333	0.600	0.537	5	0.652	0.667	0.591	4	0.583	0.613	0.557
D027	1	0.000	0.000	0.000	2	0.042	0.042	0.040	3	0.043	0.127	0.120	1	0.000	0.000	0.000
D029	3	0.500	0.563	0.478	5	0.444	0.648	0.589	4	0.400	0.572	0.460	5	0.421	0.688	0.627
D032	3	0.313	0.534	0.412	3	0.188	0.647	0.551	4	0.286	0.655	0.574	5	0.333	0.651	0.565
D033	4	0.458	0.391	0.355	5	0.375	0.336	0.315	5	0.667	0.527	0.481	4	0.375	0.442	0.395
D036	4	0.565	0.621	0.539	5	0.652	0.520	0.474	4	0.650	0.581	0.473	4	0.650	0.640	0.564
D038	5	0.739	0.645	0.585	4	0.417	0.608	0.537	4	0.958	0.712	0.647	4	0.857	0.717	0.646
D039	2	0.333	0.383	0.305	3	0.292	0.616	0.530	3	0.375	0.462	0.381	4	0.130	0.312	0.288
R007	4	0.267	0.634	0.569	5	0.063	0.671	0.597	7	0.375	0.725	0.668	5	0.200	0.774	0.694
R022	4	0.125	0.384	0.340	3	0.333	0.635	0.543***	7	0.348	0.449	0.424	4	0.190	0.339	0.313
R026	4	0.308	0.775	0.698	3	0.667	0.681	0.588	5	0.545	0.755	0.692	4	0.444	0.739	0.649
R031	5	0.364	0.407	0.371	1	0.000	0.000	0.000	2	0.000	0.102	0.095	4	0.333	0.424	0.371
R037	5	0.708	0.639	0.567	5	0.417	0.681	0.608	7	0.524	0.503	0.460	5	0.375	0.504	0.462
R043	5	0.333	0.303	0.285	4	0.708	0.516	0.440	8	0.458	0.401	0.376	8	0.458	0.464	0.439
R045	3	0.318	0.538	0.427	4	0.286	0.717	0.642	4	0.565	0.579	0.503	4	0.250	0.722	0.645
R049	3	0.125	0.331	0.294	4	0.526	0.422	0.362	6	0.476	0.655	0.590	7	0.385	0.615	0.569
R054	2	0.176	0.515	0.375	3	0.100	0.631	0.534	2	0.348	0.348	0.282	3	0.385	0.631	0.529
R056	1	0.000	0.000	0.000	3	0.083	0.082	0.079	3	0.435	0.357	0.301	2	0.043	0.043	0.042
R058	5	0.647	0.626	0.543	6	0.762	0.756	0.697	8	0.905	0.727	0.666	8	0.714	0.794	0.742
R087	6	0.833	0.738	0.679	6	0.625	0.774	0.718	7	0.875	0.758	0.708	7	0.739	0.740	0.681
R090	3	0.267	0.480	0.383	4	0.333	0.314	0.283	3	0.067	0.191	0.175	3	0.000	0.450	0.385

Note

*A* = number of alleles per locus; *H*_e_ = expected heterozygosity; *H*_o_ = observed heterozygosity; *N* = number of individuals sampled; PIC*=* polymorphism information content.

^a^Locality and voucher information are available in Appendix 1.

^b^Significant deviations from Hardy–Weinberg equilibrium: *P < 0.05, **P < 0.01, ***P < 0.001.

**Table 3:** Cross‐amplification efficiency of 33 microsatellite loci developed for *Smilax rotundifolia* in three congeneric species.^a^

Locus	*Smilax riparia* (*N* = 5)		*Smilax china* (*N* = 5)		*Smilax walteri* (*N* = 5)	
	Efficiency (%)	Allele size (bp)	Efficiency (%)	Allele size (bp)	Efficiency (%)	Allele size (bp)
D004	100	124	100	104–122	100	132–140
D005	100	188–192	100	198–204	100	168–188
D007	0	—	0	—	100	132–134
D009	0	—	0	—	100	154–166
D010	0	—	0	—	0	—
D012	100	175–181	100	173	100	152–176
D014	100	168	100	177–186	100	126–129
D016	60	177	0	—	100	189–195
D017	0	—	100	201–210	60	143–146
D021	0	—	0	—	100	142–151
D023	100	123–129	100	129–132	100	113–119
D024	0	—	0	—	0	—
D025	100	114–118	100	126–150	100	130
D027	0	—	20	138	100	130
D029	40	170	0	—	0	—
D032	100	214–242	0	—	0	—
D033	0	—	0	—	100	226–242
D036	0	—	0	—	100	136–144
D038	100	160	100	172–177	100	120–125
D039	40	220	100	250	40	235
R007	0	—	0	—	100	232
R022	100	168–174	100	180–188	100	172–174
R026	0	—	100	300–308	100	262–266
R031	100	254–260	40	220	0	—
R037	100	130	100	120–140	100	124–160
R043	100	144	100	163–169	0	—
R045	100	311–314	80	282–285	0	—
R049	0	—	0	—	0	—
R054	0	—	0	—	0	—
R056	40	116–122	40	116–118	100	116–125
R058	0	—	0	—	100	109–181
R087	100	106–112	100	124–130	100	124–172
R090	100	282–288	80	282	0	—
Transferability^b^	29/33 = 57.60%		29/33 = 57.60%		23/33 = 69.70%	

Note

— = unsuccessful amplification; *N* = number of individuals sampled.

a Locality and voucher information are available in Appendix 1.

b Successful cross‐amplification is shown as locus number/total number of microsatellite markers × 100%.

## CONCLUSIONS

In this study, we developed and characterized 33 polymorphic microsatellite markers that may prove useful in assessing genetic structure and gene flow across the geographic range of *S. rotundifolia*. The results of cross‐amplification tests of these SSR primer pairs in three congeneric *Smilax* species suggests that these markers may also be useful for assessing genetic variation and genetic structure in other *Smilax* species.

## APPENDIX 1 Locality and voucher information for populations of *Smilax rotundifolia*,* S. china*,* S. riparia*, and *S. walteri* used in this study. Voucher specimens were deposited at the herbarium of Zhejiang University (HZU), Hangzhou, Zhejiang, China.

**Table:**

Species	Population code	Voucher no.	Locality	Geographic coordinates	*N*
*Smilax rotundifolia* L.	NE	*P. Li 10042*	Holland, Michigan, USA	43°47′45″N, 86°09′04″W	24
	CE	*P. Li 161773*	Snow Hill, Maryland, USA	38°08′08″N, 75°26′27″W	24
	SE	*P. Li 150261*	Eastover, South Carolina, USA	33°53′29″N, 80°39′48″W	24
	CS	*P. Li 10125*	Winters, Texas, USA	31°58′06″N, 99°54′07″W	24
*Smilax china* L.		*Z. Qi & P. Li 160136*	Wenzhou, China	27°42′21″N, 119°40′30″E	5
*Smilax riparia* A. DC.		*Y. Chen 160344*	Hengyang, China	27°16′33″N, 112°40′42″E	5
*Smilax walteri* Pursh		*P. Li 162117*	Franklinton, Louisiana, USA	30°46′17″N, 90°09′22″W	5

Note

*N* = number of individuals sampled.
